# Factor analysis and evaluation of each item of the tinnitus handicap inventory

**DOI:** 10.1186/s13005-020-00217-3

**Published:** 2020-03-07

**Authors:** Satoko Wakabayashi, Naoki Oishi, Seiichi Shinden, Kaoru Ogawa

**Affiliations:** 1 Department of Otorhinolaryngology-Head and Neck Surgery, Keio University School of Medicine, 35 Shinanomachi, Shinjuku-ku, Tokyo, 160-8582 Japan; 2 Department of Otorhinolaryngology, Tokyo Metropolitan Hospital Ohtsuka, 2-8-1 Minamiohtsuka, Toshima-ku, Tokyo, 170-8476 Japan; 3 Department of Otorhinolaryngology, Saiseikai Utsunomiya Hospital, 911-1 Takebayashicho, Utsunomiya-shi, Tochigi, 321-0974 Japan

**Keywords:** Tinnitus, THI (tinnitus handicap inventory), Subscales, Total score, Comorbid symptoms

## Abstract

**Purpose:**

This study aims to examine the availability of subscales in the Tinnitus Handicap Inventory (THI) originally proposed by Newman and the possibility of other useful subscales. We also examine whether each item of the THI could be used to better understand the status of patients with tinnitus.

**Methods:**

This study included 1332 patients who answered the THI on their first visit. Confirmatory factor analysis was conducted to the 25 items of the THI to confirm the usefulness of the emotional, functional, and catastrophic subscales. Exploratory factor analysis was performed to discover the availability of other suitable subscales in addition to the proposed subscales. The proportion of patients who chose “yes” in each item of the THI was also examined to understand the status of patients with tinnitus.

**Results:**

In the confirmatory factor analysis, the emotional, functional, and catastrophic subscales did not fit the model. In the exploratory factor analysis, data were extremely biased to one factor. Examination of each item of the THI showed the tendency of worsening of comorbid symptoms when tinnitus handicap became worse.

**Conclusions:**

As a result of the factor analysis, only the total score, not any subscale, would be clinically useful in the THI. Examination of each item of the THI was helpful to understand the status of patients with tinnitus and comorbid symptoms of tinnitus. It is necessary to consider treatment by taking these comorbid symptoms into account.

## Background

Tinnitus Handicap Inventory (THI) is a reliable and valid questionnaire to evaluate tinnitus-related disability in patients with tinnitus [1]. Tinnitus Research Initiative (TRI), an international academic organization founded in 2006, recommended the use of THI for evaluation of tinnitus handicap and therapeutic effect [2].

The THI has been translated into many languages and used internationally. The translations include Italian [3], Brazilian Portuguese [4], Chinese (Chinese-Mandarin [5] and Chinese-Cantonese [6]), etc. Reliability and validity have been demonstrated for these translations. In Japan, the Japanese version of THI validated by Shinden et al. is used [7]. The used questionnaires to assess the construct validity differ among languages. In Italian version, the MOS (Medical Outcomes Study) 36-Item Short Form Health Survey (SF-36) [8] and the Hospital Anxiety and Depression Scale (HADS) [9] were used, while the Beck Depression Inventory (BDI) [10] was used in Brazilian Portuguese version. In Japanese version, the Self-Rating Depression Scale (SDS) [11] and the State-Trait Anxiety Inventory (STAI) [12] were used [13]. The TRI stated that differences in the culture, language, and health care system have a significant impact on tinnitus evaluation [2].

In daily medical practice, the total score of THI is mainly used, and the severity is divided based on the total score. There is a report concluded that the THI could only be discussed by the total score [14], whereas a recent report concluded that, by using factor analysis, the THI can be discussed by subscales originally indicated by Newman [15]. In Baguley’s report, data were collected from 80 patients with tinnitus and 116 patients with vestibular schwannomas awaiting surgery. In Kleinstauber’s report, data were collected from 373 patients with tinnitus who were recruited through the internet for different research.

In the present study, more than 1000 patients presented to a hospital with tinnitus were recruited, and this large amount of data were considered to be meaningful to evaluate the contradictory results. This study aims to examine the appropriateness of using the THI subscales indicated by Newman for discussion and to determine the possibility of other useful subscale apart from the total score. We also examine whether each item of the THI could be used to better understand the status of patients with tinnitus.

## Methods

The present study included a total of 1332 patients with tinnitus, 696 men (52.2%) and 636 females (47.7%), who visited the department of otolaryngology in Keio University Hospital between 1 January 2004 and 31 December 2011 and answered the THI on their first visit. The average age of the subjects was 58.7 ± 13.8 years. We used the Japanese version of THI [7]. Patients who could not complete the Japanese version of THI due to their age or language were excluded. The THI consists of 25 items. Each question item of the Japanese version of THI is listed in Table 1. The original version of THI is listed in Table 2.

**Table 1:** The Japanese version of Tinnitus Handicap Inventory

	Subscale^a^		よくある	たまにある	ない
1	F	耳鳴のために物事に集中できない。	4	2	0
2	F	耳鳴の音が大きくて人の話が聞き取れない。	4	2	0
3	E	耳鳴に対して腹が立つ。	4	2	0
4	F	耳鳴のために混乱してしまう。	4	2	0
5	C	耳鳴のために絶望的な気持ちになる。	4	2	0
6	E	耳鳴について多くの不満を訴えてしまう。	4	2	0
7	F	夜眠るときに耳鳴が妨げになる。	4	2	0
8	C	耳鳴から逃れられないかのように感じる。	4	2	0
9	F	あなたの社会的活動が耳鳴により妨げられている。(例えば、外食をする、映画を観るなど)	4	2	0
10	E	耳鳴のために挫折を感じる。	4	2	0
11	C	耳鳴のために自分がひどい病気であるように感じる。	4	2	0
12	F	耳鳴があるために日々の生活を楽しめない。	4	2	0
13	F	耳鳴が職場や家庭での仕事の妨げになる。	4	2	0
14	E	耳鳴のためにいらいらする。	4	2	0
15	F	耳鳴のために読書ができない。	4	2	0
16	E	耳鳴のために気が動転する。	4	2	0
17	E	耳鳴のために家族や友人との関係にストレスを感じる。	4	2	0
18	F	耳鳴から意識をそらすのは難しいと感じる。	4	2	0
19	C	自分一人で耳鳴を管理していくのは難しいと感じる。	4	2	0
20	F	耳鳴のために疲れを感じる。	4	2	0
21	E	耳鳴のために落ち込んでしまう。	4	2	0
22	E	耳鳴のために体のことが心配になる。	4	2	0
23	C	耳鳴とこれ以上つき合っていけないと感じる。	4	2	0
24	F	ストレスがあると耳鳴がひどくなる。	4	2	0
25	E	耳鳴のために不安な気持ちになる。	4	2	0

^a^F represents an item contained on the functional subscale; E, an item contained on the emotional subscale; and C, an item contained on the catastrophic subscale

**Table 2:** The original version of Tinnitus Handicap Inventory

	Subscale^a^		Yes	Sometimes	No
1	F	Because of your tinnitus, is it difficult for you to concentrate?	4	2	0
2	F	Does the loudness of your tinnitus make it difficult for you to hear people?	4	2	0
3	E	Does your tinnitus make you angry?	4	2	0
4	F	Does your tinnitus make you feel confused?	4	2	0
5	C	Because of your tinnitus, do you feel desperate?	4	2	0
6	E	Do you complain a great deal about your tinnitus?	4	2	0
7	F	Because of your tinnitus, do you have trouble falling to sleep at night?	4	2	0
8	C	Do you feel as though you cannot escape your tinnitus?	4	2	0
9	F	Does your tinnitus interfere with your ability to enjoy your social activities (such as going out to dinner, to the movies)?	4	2	0
10	E	Because of your tinnitus, do you feel frustrated?	4	2	0
11	C	Because of your tinnitus, do you feel that you have a terrible disease?	4	2	0
12	F	Does your tinnitus make it difficult for you to enjoy life?	4	2	0
13	F	Does your tinnitus interfere with your job or household responsibilities?	4	2	0
14	E	Because of your tinnitus, do you find that you are often irritable?	4	2	0
15	F	Because of your tinnitus, is it difficult for you to read?	4	2	0
16	E	Does your tinnitus make you upset?	4	2	0
17	E	Do you feel that your tinnitus problem has placed stress on your relationships with members of your family and friends?	4	2	0
18	F	Do you find it difficult to focus your attention away from your tinnitus and on other things?	4	2	0
19	C	Do you feel that you have no control over your tinnitus?	4	2	0
20	F	Because of your tinnitus, do you often feel tired?	4	2	0
21	E	Because of your tinnitus, do you feel depressed?	4	2	0
22	E	Does your tinnitus make you feel anxious?	4	2	0
23	C	Do you feel that you can no longer cope with your tinnitus?	4	2	0
24	F	Does your tinnitus get worse when you are under stress?	4	2	0
25	E	Does your tinnitus make you feel insecure?	4	2	0

^a^F represents an item contained on the functional subscale; E, an item contained on the emotional subscale; and C, an item contained on the catastrophic subscale

In the THI, scores of 0, 2, or 4 are assigned to each answer, and thus the total score ranges from 0 to 100. Higher THI scores indicate a greater handicap from tinnitus, and five categories are used: (i) no handicap (0–16), (ii) mild handicap (18–36), (iii) moderate handicap (38–56), (iv) severe handicap (58–76), and (v) catastrophic handicap (78–100) [16]. The three subscales indicated by Newman: functional, emotional, and catastrophic were included. The distribution of each question to subscales is described in Tables 1 and 2.

Confirmatory factor analysis was conducted to the 25 items of the THI to confirm the usefulness of the three proposed subscales. Subsequently, exploratory factor analysis was performed to discover the availability of other suitable subscales in addition to the proposed subscales. Besides, the proportion of patients who chose “yes” (score 4) in each THI item was examined in each severity. We picked up items if the proportion of the patients who chose “yes” exceeded 10%, and examined the tendency of which items increased when tinnitus became severe.

For statistical analysis, SPSS Statistics 24 (IBM, New York, NY, USA) was used for calculation of average value and exploratory factor analysis, and SPSS Amos 24 (IBM, New York, NY, USA) for confirmatory factor analysis. Statistical values were considered significant at 5% level.

## Results

The average THI total score on the first visit was 54.2 ± 26.0. The tinnitus severity distributions of the subjects were 8.9% with no handicap (*n* = 119), 19.9% with mild handicap (*n* = 265), 24.1% with moderate handicap (*n* = 321), 22.7% with severe handicap (*n* = 303), and 24.3% with catastrophic handicap (*n* = 324).

### Confirmatory factor analysis

**Fig. 1:**
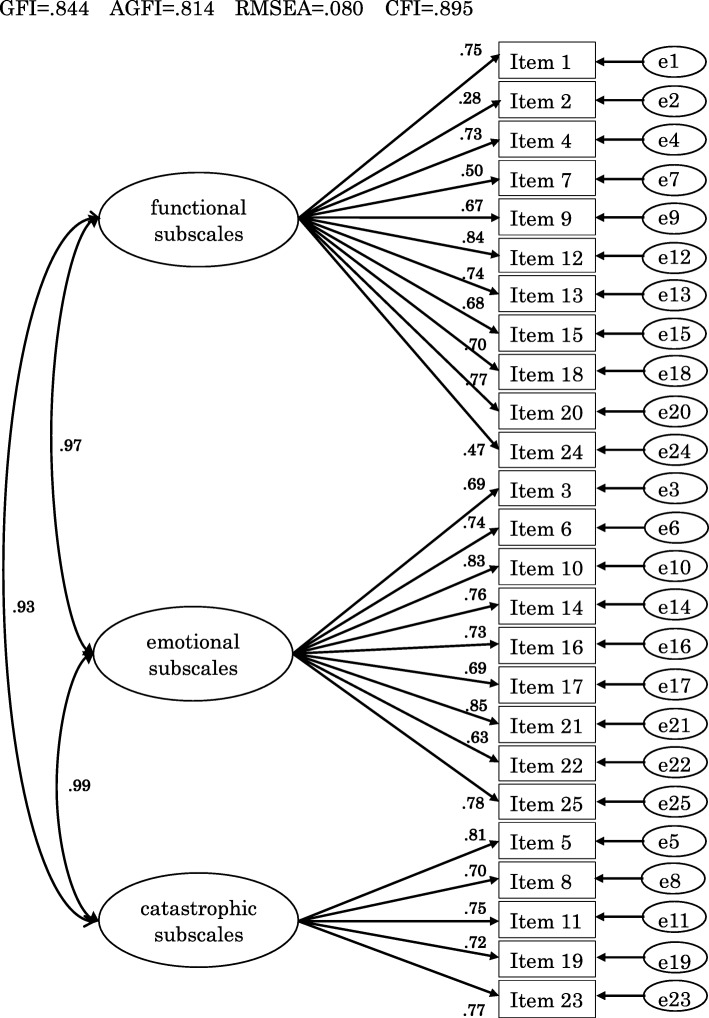
Path diagram of three-factor model of the THI. As indicators of model fitness, Good of Fit Index (GFI), Adjusted GFI (AGFI), Root Mean Square Error of Approximation (RMSEA), and Comparative Fit Index (CFI) are used. GFI, AGFI, and CFI should be 0.95 or higher and RMSEA 0.06 or lower

A three-factor model was set using the three subscales and confirmatory factor analysis was conducted. Results are shown in Fig. 1. Next, Good of Fit Index (GFI), Adjusted GFI (AGFI), Root Mean Square Error of Approximation (RMSEA), and Comparative Fit Index (CFI) were used as indicators of model fitness. GFI, AGFI, and CFI should be 0.95 or higher and RMSEA 0.06 or lower [17, 18]. As a result, in the three-factor model of THI, none of the criteria were met, i.e., GFI = 0.844, AGFI = 0.814, RMSEA = 0.080, CFI = 0.895. Besides, the correlation between the three factors was very high. These results suggested that the three subscales did not fit the model.

### Exploratory factor analysis

**Fig. 2:**
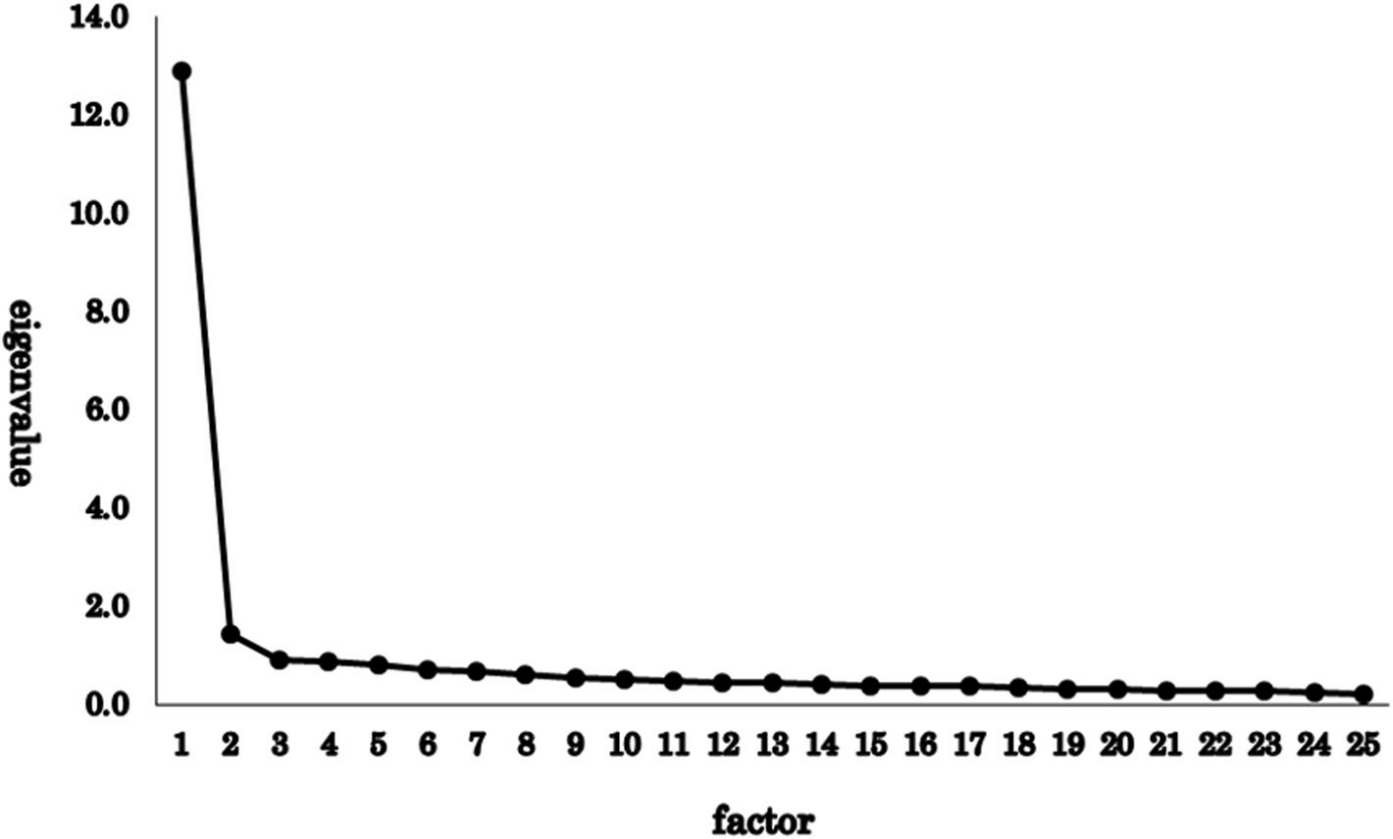
Scree plot of the exploratory factor analysis for 25 items of THI. The eigenvalues for the factors were 12.88 (factor 1) and 1.45 (factor 2), explaining 57.3% of the variance

For the 25 items of THI, we used maximum likelihood method and promax rotation method to perform exploratory factor analysis. The scree plot is shown in Fig. 2, and the factor matrix is shown in Table 3. The data were extremely biased to one factor, and there were only two factors of which eigenvalue exceeded one. The eigenvalues for the factors were 12.88 (Factor 1) and 1.45 (Factor 2), explaining 57.3% of the variance. Adding more factors contributed little variance, with Factor 3 adding 3.6% (eigenvalue = 0.91) and Factor 4 adding 3.4% (eigenvalue = 0.86). The correlation between Factor 1 and Factor 2 was very high (0.77), and in the goodness of fit test, the result suggested that the factors did not fit the model (Χ^2^ = 1660.89, df = 251, *p* = 0.000).

**Table 3:** Factor loadings of exploratory factor analysis (loadings above 0.40 are presented)

Item	Factor 1	Factor 2
1	0.72	
2		0.39
3	0.68	
4	0.74	
5	0.8	
6	0.74	
7	0.5	
8	0.69	
9	0.65	
10	0.82	
11	0.73	
12	0.83	
13	0.72	
14	0.76	
15	0.66	
16	0.73	
17	0.71	
18	0.7	
19	0.72	
20	0.76	
21	0.85	
22	0.62	
23	0.75	
24	0.47	
25	0.77	

These results indicated that only the total score, not any subscale, would be clinically useful.

### Each item of the THI

**Fig. 3:**
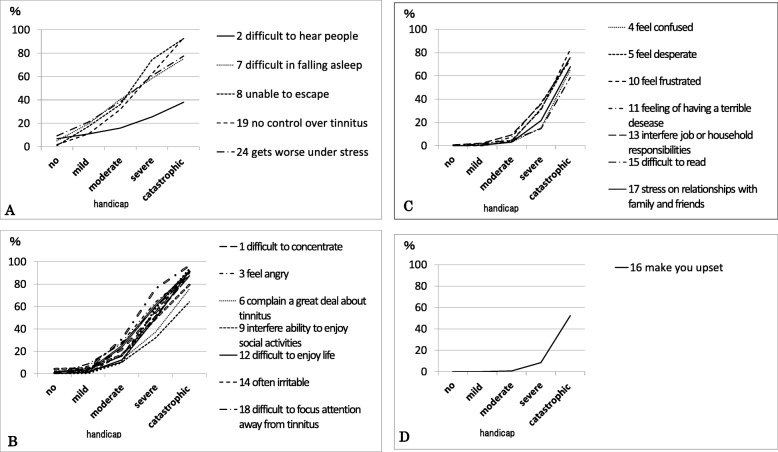
Changes in the proportion of the patients who chose “yes” in each item. The graphs are divided according to the tinnitus handicap severity of which more than 10% of patients chose “yes”. **A**: Five items were chosen as ‘yes’ by more than 10% of the ‘mild handicap’ patients. **B**: Twelve items were chosen as ‘yes’ by more than 10% of the ‘moderate handicap’ patients. **C**: Seven items were chosen as ‘yes’ by more than 10% of the ‘severe handicap’ patients. **D**: One item was chosen as ‘yes’ by more than 10% of the ‘catastrophic handicap’ patients

Items being selected “yes” by more than 10% of the subjects were chosen for plotting a graph for each severity category of the THI (Fig. 3A, B, C, D). In the group of no handicap, there was no item whose proportion of being selected “yes” exceeded 10%. The item that was selected “yes” by the largest proportion in the group of no handicap was item 24: “Does your tinnitus get worse when you are under stress?,” and the proportion was 9.2%.

In the group of mild handicap, 5 items were selected “yes” by more than 10% of the subjects. They were as follows:

(i) item 2: “Does the loudness of your tinnitus make it difficult for you to hear people?”;
(ii) item 7: “Because of your tinnitus, do you have trouble falling to sleep at night?”;
(iii) item 8: “Do you feel as though you cannot escape your tinnitus?”;
(iv) item 19: “Do you feel that you have no control over your tinnitus?”; and
(v) item 24: “Does your tinnitus get worse when you are under stress?,”

These indicate that these symptoms are observed in relatively mild cases. Figure 3A shows the change of the proportion of being selected “yes” in each tinnitus severity for these five items. Difficulty in listening, as suggested from item 2, was relatively high in mild handicap patients, whereas this symptom was relatively low in the catastrophic handicap patients.

In the group of moderate handicap, other 12 items were selected “yes” by more than 10% of the subjects. The items were as follows:

(i) item 1: “Because of your tinnitus, is it difficult for you to concentrate?”;
(ii) item 3: “Does your tinnitus make you angry?”;
(iii) item 6: “Do you complain a great deal about your tinnitus?”;
(iv) item 9: “Does your tinnitus interfere with your ability to enjoy your social activities?”;
(v) item 12: “Does your tinnitus make it difficult for you to enjoy life?”;
(vi) item 14: “Because of your tinnitus, do you find that you are often irritable?”;
(vii) item 18: “Do you find it difficult to focus your attention away from your tinnitus and on other things?”;
(viii) item 20: “Because of your tinnitus, do you feel tired?”;
(ix) item 21: “Because of your tinnitus, do you feel depressed?”;
(x) item 22: “Does your tinnitus make you feel anxious?”;
(xi) item 23: “Do you feel that you can no longer cope with your tinnitus?”; and
(xii) item 25: “Does your tinnitus make you feel insecure?.”

Figure 3-B shows the change of the proportion of being selected “yes” in each tinnitus severity for these 12 items. These results suggested that in moderate handicap patients, the influence of tinnitus on social activities, daily life, and work has increased. It is also suggested that the proportion of admitting psychological discontent, irritation, fatigue, depression, and anxiety has increased.

In the group of severe handicap, almost all of the remaining items were selected “yes” by more than 10% of the subjects. They were as follows:

(i) item 4: “Does your tinnitus make you feel confused?”;
(ii) item 5: “Because of your tinnitus, do you feel desperate?”;
(iii) item 10: “Because of your tinnitus, do you feel frustrated?”;
(iv) item 11: “Because of your tinnitus, do you feel that you have a terrible disease?”;
(v) item 13: “Does your tinnitus interfere with your job or household responsibilities?”;
(vi) item 15: “Because of your tinnitus, is it difficult for you to read?”; and
(vii) item 17: “Do you feel that your tinnitus problem has placed stress on your relationships with members of your family and friends?.”

Figure 3-C shows the change of the proportion of being selected “yes” in each tinnitus severity for these 7 items. In the group of severe handicap, the proportion of “yes” exceeded 50% in 12 items such as not being able to concentrate (item 1), getting angry (item 3), sleep disorder (item 7), and depression (item 21). It is suggested that in severe handicap patients, many patients feel frustrated and feel that they have serious illness and human relationship failure in their daily life.

In the group of catastrophic handicap, the proportion of patients who chose “yes” newly exceeded 10% in only one item. That was as follows:

(i) item 16: “Does your tinnitus make you upset?”.

There was no item whose proportion of being selected “yes” was 10% or less in the group of catastrophic handicap. The proportion of patients who select “yes” in item 16 rise drastically in the group of catastrophic handicap (52.2%) compared with in the group of severe handicap (8.3%). Figure 3-D shows the change in the proportion of being selected “yes” in each tinnitus severity for item 16. In the group of catastrophic handicap, the proportion of “yes” exceeded 50% in nearly all item. Only item 2: “Does the loudness of your tinnitus make it difficult for you to hear people?” showed a relatively low proportion, 38%.

## Discussion

As a result of factor analysis in this study, it was considered that evaluation based on the total score was the most appropriate for the THI, as indicated by Baguley [14]. By examining individual items of the THI, it was suggested that the tendency of how tinnitus became severe could be understood.

We examined the proportion of patients with tinnitus who selected “yes” in each item of THI on their first visit to our hospital and found the tendency of symptoms which patients experienced as their tinnitus became severe. The symptoms of sleep disorders and difficulty in hearing were often seen in relatively mild cases. Their work, social lives, and daily lives were disturbed by tinnitus in moderate cases. In a psychological aspect, the patients were not able to enjoy their lives and tended to feel fatigue, depression, and anxiety. As tinnitus became severe, many patients felt despair, frustration, and severe illness, and human relations also interfered.

Sleep disorders, depression, anxiety have been reported to be related to tinnitus [13, 19, 20]. In this study, it became clear that patients with tinnitus are often bothered with sleep disorders, even if the tinnitus handicap is relatively mild. This suggests that management of sleep problems is important at the early stages of tinnitus. In our previous study, sleep disorder was observed in 70% of all patients with tinnitus [21]. In the previous study, there was no clear relationship between the presence or absence of sleep disorder and severity of tinnitus, which is consistent with the results obtained from each THI item in this study.

As tinnitus handicap becomes moderate, more patients suffer from depressive symptoms and anxiety symptoms. Although the THI can easily evaluate severity in a convenient manner, using only THI is insufficient to grasp details of comorbid symptoms such as depressive and anxiety symptoms. In the TRI, for evaluation of the status of patients and the therapeutic effect, some questionnaires are mentioned such as the BDI (Beck Depression Inventory [10]), the STAI (State-Trait Anxiety Inventory [12]), the PSQI (Pittsburgh Sleep Quality Index [22]) [2]. The recommendation degree of these questionnaires is C: might be of interest, but considering that the proportion of patients with depression and anxiety symptoms is higher in patients with tinnitus of moderate or severe handicap, these questionnaires should be useful.

In patients with severe and catastrophic handicap, there are many patients who feel despair, frustration, and severe illness, requiring both physical and mental care. These results suggest that in the treatments of patients with severe and catastrophic tinnitus handicap who show symptoms of despair, frustration, and severe illness, it is important to cooperate with psychiatrists or psychologists proactively.

Also, the percentage of patients who selected “yes” to item 2: “Does the loudness of your tinnitus make it difficult for you to hear people?” was more than 10% in mild handicap patients, whereas it did not exceed 40% in catastrophic handicap patients. In the catastrophic handicap patients, the proportion of patients who selected “yes” in this item 2 was overwhelmingly low compared with the other question items. As a treatment of tinnitus, the use of a hearing aid is recommended when hearing loss is accompanied, and its effectiveness has been reported [23, 24]. However, based on our results, the use of hearing aids alone might not be efficient to treat patients with catastrophic tinnitus, and hence psychiatric treatment should be considered.

Our results show that evaluation based on the total score is appropriate for THI, which differs from the report by Kleinstauber [15]. In Kleinstauber’s study, patients with tinnitus were part of those participating in their cognitive behavior therapy recruited through the Internet. The average total score of THI was lower than our research (41.3 in the Kleinstauber’s study; and 54.2 in this study). In our study, the participants were the patients who visited us for treatment as chief complaints of tinnitus. As shown in the path diagram (Fig. 1), the correlation between factors is very strong, which is higher than that reported by Kleinstauber. By item, the path coefficients of item 8 (Do you feel as though you cannot escape your tinnitus?) and item 19 (Do you feel that you have no control over your tinnitus?) was significantly different from Kleinstauber’s report. These items are included in the catastrophic subscale proposed by Newman, but in our study, these items were selected “yes” by relatively high proportion of patients with mild tinnitus handicap. Differences in these results may be related to different backgrounds including cultural difference.

In this study, we collected data from as many as 1332 patients. We found that symptoms such as sleep disorder and difficulty in hearing are relatively frequently seen in mild tinnitus handicap patients. When tinnitus handicap becomes moderate, the proportion of patients who feel tired, depression, anxiety, difficulty in enjoying life, and so on, increased. As tinnitus handicap becomes severe, many patients feel desperate, frustrated, severe illness, and human relations are disturbed. These changes are important in grasping the patient’s condition and choosing treatment.

## Conclusion

As a result of the factor analysis, the THI can be used as a unifactorial measurement of tinnitus handicap, and it is most appropriate to evaluate based on the total score.

By examining each item of the THI, we could observe which comorbid symptoms will frequently appear when tinnitus handicap becomes severe. It is necessary to choose treatment by considering that these comorbid symptoms emerge when tinnitus handicap becomes severe.

## Data Availability

The datasets used and/or analysed during the current study are available from the corresponding author on reasonable request.

## Abbreviations

AGFI: Adjusted Good of Fit Index
BDI: Beck Depression Inventory
CFI: Comparative Fit Index
GFI: Good of Fit Index
HADS: Hospital Anxiety and Depression Scale
MOS: Medical Outcomes Study
PSQI: Pittsburgh Sleep Quality Index
RMSEA: Root Mean Square Error of Approximation
SDS: Self-Rating Depression Scale
SF-36: 36-Item Short Form Health Survey
STAI: State-Trait Anxiety Inventory
THI: Tinnitus Handicap Inventory
TRI: Tinnitus Research Initiative
